# Reliability of Spectral Features of Resting-State Brain Activity: A Magnetoencephalography Study

**DOI:** 10.7759/cureus.52637

**Published:** 2024-01-20

**Authors:** Eiichi Okumura, Hideyuki Hoshi, Hirofumi Morise, Naohiro Okumura, Keisuke Fukasawa, Sayuri Ichikawa, Takashi Asakawa, Yoshihito Shigihara

**Affiliations:** 1 Medical Imaging Business Center, Ricoh Company, Ltd., Kanazawa, JPN; 2 Precision Medicine Centre, Hokuto Hospital, Obihiro, JPN; 3 Precision Medicine Centre, Kumagaya General Hospital, Kumagaya, JPN

**Keywords:** magnetoencephalography, reliability, reproducibility, minimal detectable change, resting-state, frequency analysis, spectral parameter

## Abstract

Background

Cognition is a vital sign and its deterioration is a major concern in clinical medicine. It is usually evaluated using neuropsychological assessments, which have innate limitations such as the practice effect. To compensate for these assessments, the oscillatory power of resting-state brain activity has recently become available. The power is obtained noninvasively using magnetoencephalography and is summarized by spectral parameters such as the median frequency (MF), individual alpha frequency (IAF), spectral edge frequency 95 (SEF95), and Shannon’s spectral entropy (SSE). As these parameters are less sensitive to practice effects, they are suitable for longitudinal studies. However, their reliability remains unestablished, hindering their proactive use in clinical practice. Therefore, we aimed to quantify the within-participant reliability of these parameters using repeated measurements of healthy participants to facilitate their clinical use and to evaluate the observed changes/differences in these parameters reported in previous studies.

Methodology

Resting-state brain activity with eyes closed was recorded using magnetoencephalography for five minutes from 15 healthy individuals (29.3 ± 4.6 years old: ranging from 23 to 28 years old). The following four spectral parameters were calculated: MF, IAF, SEF95, and SSE. To quantify reliability, the minimal detectable change (MDC) and intraclass correlation coefficient (ICC) were computed for each parameter. In addition, we used MDCs to evaluate the changes and differences in the spectral parameters reported in previous longitudinal and cross-sectional studies.

Results

The MDC at 95% confidence interval (MDC95) of MF, IAF, SEF95, and SSE were 0.61 Hz, 0.44 Hz, 2.91 Hz, and 0.028, respectively. The ICCs of these parameters were 0.96, 0.92, 0.94, and 0.83, respectively. The MDC95 of these parameters was smaller than the mean difference in the parameters between cognitively healthy individuals and patients with dementia, as reported in previous studies.

Conclusions

The spectral parameter changes/differences observed in prior studies were not attributed to measurement errors but rather reflected genuine effects. Furthermore, all spectral parameters exhibited high ICCs (>0.8), underscoring their robust within-participant reliability. Our results support the clinical use of these parameters, especially in the longitudinal monitoring and evaluation of the outcomes of interventions.

## Introduction

Cognitive impairment is a major concern in clinical medicine [1]. The level of impairment is usually evaluated using neuropsychological assessments, such as the Mini-Mental State Examination (MMSE) [2]. However, neuropsychological assessments have intrinsic limitations, such as the practice effect [3], which biases the results when used repeatedly. This reduces the reliability of repeated assessments in longitudinal studies. Therefore, alternative assessment methods are required.

Recently, magnetoencephalography (MEG) has been used as a clinical tool to assess cognitive impairment levels and compensate for neuropsychological assessment limitations [4]. It non-invasively records resting-state brain activity in terms of oscillatory power, and its spectral features reflect cognitive impairment level. The spectral features are summarised objectively using spectral parameters such as median frequency (MF), individual alpha frequency (IAF), spectral edge frequency 95 (SEF95), and Shannon’s spectral entropy (SE). Unlike neuropsychological assessments or classical visual inspections of electrophysiological waveforms, these parameters are computed mechanically without the subjective interpretation of raw data. Furthermore, these parameters are less sensitive to practice effects [5]. Although previous studies have demonstrated their clinical utility [6], it remains unclear how much these parameters fluctuate across repeated measurements within the same participant (i.e., within-participant variability).

The minimal detectable change (MDC) is an index that represents the reliability of measurements in science and technology [7] and is used to estimate the random measurement error of the parameters. MDC is an absolute reliability measure as it retains the measurement unit of the parameters being evaluated (e.g., Hz for MF), which allows us to directly compare the effects of interest (e.g., longitudinal changes such as pre- vs. post-intervention and follow-up periods, or cross-sectional differences between diagnostic groups) and MDC. Importantly, effects larger than MDC are deemed significant. For instance, in the rehabilitation field, MDC has been employed to assess the efficacy of interventions [8], with effectiveness determined when changes in parameters that quantify symptoms (i.e., the effect of interest) exceed the MDC. In our study, we applied the MDC concept to cognitive impairment, using spectral parameters. Effects are considered meaningful if spectral parameters (i.e., MF, IAF, SEF95, and SSE) exhibit changes or differences exceeding their respective MDCs. Thus, we utilized MDCs to evaluate spectral parameter effects reported in prior studies. While MDCs serve as valuable reliability measures for assessing effects, their uniqueness to each parameter’s measurement unit precludes cross-parameter comparisons. To address this limitation, we also introduced the intraclass correlation coefficient (ICC), a relative reliability measure enabling the comparison of reproducibility between parameters due to its normalized unit representation [9].

In this study, we investigated the within-participant reliabilities of spectral parameters (MF, IAF, SEF95, and SSE) using MDC and ICC based on resting-state MEG data from cognitively healthy (CH) individuals. Moreover, we evaluated the effects of interest in these parameters, as reported in previous studies, using MDCs. The preceding studies were systematically selected for this evaluation, and their details are presented in a systematic review format in the following sections. Therefore, this article adopts a dual, hybrid approach, combining elements of a research article (to investigate the within-participant reliability of the spectral parameters) and a systematic review (to appraise earlier findings using the reliability measures).

## Materials and methods

Participants

Fifteen healthy young participants (11 females and four males) between the ages of 23 and 38 (mean ± standard deviation (SD) = 29.3 ± 4.6 years) were recruited between August 2023 and October 2023. None of the patients had a history of neurological, psychiatric, or other medical conditions that affect the central nervous system. This study was conducted in accordance with the ethical principles of the Declaration of Helsinki and was approved by the Ethics Committee of Kumagaya General Hospital (approval number: #13). All participants provided written informed consent to participate in the study.

MEG recording

Resting-state cortical activity was recorded for five minutes per session using a whole-head-type MEG system (RICOH160-1; Ricoh Company, Ltd., Tokyo, Japan) equipped with 160-channel axial gradiometers and placed in a magnetically shielded room at Kumagaya General Hospital, Japan. The sensor coils were gradiometers, each with 15 mm diameter and 50 mm height. The pairs of sensor coils were separated by a distance of 23 mm. The sampling frequency was 2,000 Hz with 500 Hz low-pass filtering during the recording. During the scan, participants were asked to remain awake and calm in the supine position with their eyes closed. To maintain an optimal state of vigilance, the recording was initiated shortly after closing the door of the magnetically shielded room, followed by a reminder via the intercom for the participants to stay awake with their eyes closed. Each participant completed three sessions. Between sessions, they moved out of the MEG dewer and sat upright in the MEG room, where they counted 10 seconds by themselves to restore their state of vigilance.

Pre-processing of MEG data

MEG data were analyzed offline using RICOH MEG Analysis software (Ricoh, Tokyo, Japan), MATLAB (MathWorks, MA, USA), and MATLAB custom scripts. First, a 50 Hz band-stop filter using finite impulse response filtering with a Hamming window was applied to the continuous MEG signal to remove power line noise. Next, continuous MEG signals were cleaned using a dual-signal subspace projection (DSSP) algorithm [10] available in the vendor-provided software (RICOH MEG Analysis), which is comparable to the temporally extended signal-space separation algorithm. To remove the remaining artifacts, the signals were decomposed via independent component analysis (ICA) using the Picard algorithm [11]. Each ICA component was visually inspected, and those for cardiac, blinking, saccades, and other salient artifacts were rejected. The signals were then filtered using finite impulse response filtering with a Hamming window by applying a band-pass filter (1-70 Hz). Next, the MEG sensor data were divided into non-overlapping 10-second segments (hereafter referred to as trials).

Next, to control for data quality, trials with low arousal were rejected using a modified version of the algorithm used in a previous study [12]. The algorithm was based on the physiological findings that alpha amplitudes decrease and alpha waves become unclear during drowsiness [13,14], which are atypical for healthy adult individuals in an awake state. In the original algorithm, for removing these atypical data from electroencephalography (EEG) signals, it computed the spectral powers at the central and peripheral frequencies of the alpha, theta, and beta powers (alpha = 9-11 Hz (central), 8-12 Hz (peripheral); theta = 4-5 Hz (central), 3 and 6-7 Hz (peripheral); beta = 20-22 Hz (central), 17-19 and 23-25 Hz (peripheral)] from an average power spectral density (PSD) across all channels for each trial. The retained trials had higher spectral powers at the central frequencies than those in peripheral frequencies in all three frequency bands [12]. However, in our dataset, we sometimes observed the effect of µ-rhythms on the PSD, which enhanced the alpha peak prominently in the central-parietal regions. To handle this effect, we modified the algorithm as follows: (1) the PSD was averaged across 30 occipital sensors instead of all sensors, where the physiological alpha amplitude was most prominent, and (2) we assessed alpha power alone (but not theta and beta powers) with an extended frequency range with a central frequency of 8-13 Hz and peripheral frequency of 6-7 Hz and 14-15 Hz. This modified method is referred to as the alpha peak-based trial rejection (APTR) throughout this article. One participant with fewer than 10 remaining trials after APTR was excluded from the reliability analysis. For the other participants, the remaining trials (27.7 ± 4.4 trials, maximum of 30 trials) after APTR were used in the reliability analysis.

MEG spectral parameters

Four spectral parameters in the sensor space were calculated to summarize the different characteristics of the resting-state brain activity. First, for each trial and channel, the PSD was estimated from the Fourier transform of the autocorrelation function, which corresponds to the Blackman-Tukey method [15]. The PSD was then normalized between 1 and 70 Hz (PSDn) [15], and four parameters were computed. (1) MF is the median value of the power distribution represented by PSDn and is defined as the frequency that divides the power into two halves [16]. (2) IAF represents the dominant frequency corresponding to the peak of PSDn in the alpha band and is defined similarly to the MF, except that the frequency range is adjusted to 4-15 Hz (i.e., the extended alpha band) to obtain a robust estimator of the dominant alpha oscillations [16]. (3) SEF95 is defined as the frequency that separates the total power of PSDn into the low- and high-frequency parts as 95:5 [16], which is similar to the MF but reflects the changes in the high-frequency powers more sensitively than the ones in the low frequency. (4) SSE was defined by applying the definition of normalized Shannon’s entropy to PSDn, which can be assimilated as its probability density function [16]. The SSE represents an irregularity measure closely related to the concept of order in information theory, which quantifies the homogeneity in the distribution of the oscillatory components of the PSDn. The spectral parameters averaged across the channels and trials were used to calculate the MDCs.

Reliability analyses

First, the MDC was computed for each spectral parameter. The MDC indicates a range of probability distributions of random measurement errors; therefore, it is equivalent to the confidence interval (CI) of the given measurement error. According to the range of the CIs (e.g., 95%, 90%, and 80%), different MDC measures can be computed. For example, MDC at 95% CIs (MDC95) represents the range of probability distribution in which 95% of reported measurements exist, considering the measurement error. When the differences between the two measurements were larger than MDC95, the observed difference was located outside of the 95% CI of the measurement error; thus, the null hypothesis (no differences between observed difference and measurement error) could be rejected with an alpha level of 0.05. In this study, three MDCs were calculated from the standard error of measurement (SEM) as follows [7]:

$$MDC95=SEM\times 1.96\times \sqrt{2}$$

$$MDC90=SEM\times 1.64\times \sqrt{2}$$

$$MDC80=SEM\times 1.28\times \sqrt{2}$$

Here, 1.96, 164, and 1.28 are the z values of the 95%, 90%, and 80% CIs, respectively; and $\sqrt{2}$ is multiplied to account for the additional uncertainty introduced by using repeated measurements of the parameters at two points in time. If any known or controllable factors influence the SEM, the effects can be compensated for by estimating the SEM as a residual (e.g., mean square error (MSE)) of regression models that subject the measurement with the controllable factors as predictors. In this study, we considered participants’ profiles (i.e., between-participant variance) as controllable factors [17], which should be considered when computing the SEM. Therefore, we define SEM as the square root of the MSE of a linear mixed-effects model [18].

$$SEM=\sqrt{MSE}$$

For the linear mixed-effects model (i.e., the hierarchical linear model), each spectral parameter was subjected to the function of the participants’ profiles, which is described as follows:

$$y_{ij}=\left ( \beta_{0} + b_{0i} \right )+\beta_{1} x_{1}+\beta_{2} x_{2} + \beta_{3} x_{1} x{2} + \epsilon_{ij}$$

where $y_{ij}$ is the spectral parameter in the repeated measurements over time at time $j$ (=0, 1, ..., K) for participant $i$ (=1, 2, ..., N), $\beta_{0}$ is a global intercept across all measurements, whereas $b_{0i}$ represents a random intercept estimated for participant $i$. $x_{1}$ is a continuous predictor of participants’ age and $x_{2}$ is a categorical predictor of gender. Taken together, the mixed-effects model included a fixed intercept, fixed effects of age, gender, and their interaction, and a random intercept for participants. $\epsilon_{ij}$, a residual of the measurement, was used for computing the MSE. In other words, the following equation is assumed to satisfy:

$$\epsilon_{ij}\sim N \left ( 0, \sigma^{2}_{E} \right ), b_{0i}\sim N \left ( 0, \sigma^{2}_{B0} \right ), \epsilon_{ij} \perp b_{0i}$$

This model can be expressed using Wilkinson’s notation as follows:

$$y\sim 1+age+gender+age:gender+(1|participant)$$

Second, ICC was computed for each spectral parameter. ICC is a widely used reliability index in test-retest and intra- and inter-rater reliability analyses. Mathematically, ICC represents a ratio of true variance over true variance plus error variance and reflects both the degree of correlation and the agreement between repeated measurements [19]. ICC values range between 0 and 1, with values closer to 1 representing stronger reliability. ICC values are interpreted as follows: ICC < 0.5 indicates poor reliability, 0.5 ≤ ICC < 0.75 suggests moderate reliability, 0.75 ≤ ICC < 0.9 denotes good reliability, and ICC ≥0.90 signifies excellent reliability [19]. The formula for ICC in the random intercept model is as follows [20]:

$ICC(1,K) = \frac{\sigma^{2}_{B0}}{\sigma^{2}_{B0}+\sigma^{2}_{E}}$

This reflects the proportion of error variation attributable to differences in group variables ($participants)$ relative to the total error variation of the model.

Evaluations of previous studies using MDCs

The database MEDLINE (through PubMed) was searched on September 14, 2023. All searches were repeated before the final analysis on December 13, 2023. No historical limit was applied, and no filters for study type were used. We conducted a search using the following Boolean operators: (eeg or meg) and (resting or spontaneous or rest) and (“median frequency” or “mean frequency” or “individual alpha frequency” or “peak frequency” or “dominant frequency” or “spectral edge frequency” or “Shannon’s spectral entropy” or “Shannon’s entropy”). The inclusion criteria were (1) published, peer-reviewed studies; (2) studies calculating spectral parameters (MF, IAF, SEF95, or SSE) from M/EEG data recorded during resting state in humans; (3) longitudinal or cross-sectional studies involving patients with cognitive impairment; (4) for longitudinal studies, description of the representative values (e.g. mean) of spectral parameters for each measurement; and (5) for cross-sectional studies, presentation of mean or median values and either the 95% CI, standard deviation (SD), standard error (SE), or interquartile range for each group, including CH individuals. The exclusion criteria were (1) review articles, case reports, or preprints; (2) studies not describing the representative values (e.g., mean) of spectral parameters; and (3) cross-sectional studies comparing disease groups without including CH individuals. For cross-sectional studies, we obtained group mean and SD/SE/95% CI or group median and interquartile range for each study. SDs were converted to SEs by dividing by the square root of the number of participants. SEs were converted to 95% CI by multiplying by 1.96. This data was visually represented in a figure to compare group-level differences to MDCs.

## Results

MDC

The MDCs of the MF and IAF were smaller than 1 Hz. The MDC95, MDC at 90% CIs (MDC90), and MDC at 80% CIs (MDC80) values for the MF were 0.61, 0.51, and 0.40 Hz, respectively. The MDC95, MDC90, and MDC80 values of IAF were 0.44, 0.37, and 0.29 Hz, respectively. The MDCs of SEF95 were approximately 2.5 Hz, that is, the MDC95, MDC90, and MDC80 of SEF95 were 2.91, 2.44, and 1.90 Hz, respectively. The MDCs of the SSE were less than 0.03, and the MDC95, MDC90, and MDC80 of the SSE were 0.028, 0.023, and 0.018, respectively. These values are shown in Table 1.

**Table 1:** Within-participant variabilities of spectral parameters in terms of MDC and ICC. MDC = minimal detectable change; MDC95 = MDC at 95% confidence interval; MDC90 = MDC at 90% confidence interval; MDC80 = MDC at 80% confidence interval; ICC = intraclass correlation coefficient; MF = median frequency; IAF = individual alpha frequency; SEF95 = spectral edge frequency 95; SSE = Shannon’s spectral entropy

Reliability index	MF (Hz)	IAF (Hz)	SEF95 (Hz)	SSE
MDC95	0.607	0.442	2.912	0.028
MDC90	0.508	0.370	2.436	0.023
MDC80	0.396	0.289	1.901	0.018
ICC	0.962	0.916	0.935	0.827

ICC

ICCs were >0.8 for all spectral parameters. The ICCs of MF, IAF, SEF95, and SSE were 0.96, 0.92, 0.94, and 0.83, respectively (Table 1).

Evaluations of previous studies using MDCs

The search produced a total output of 381 articles. Upon screening the articles considering only peer-reviewed journals and imposing the eligibility criteria, a total of 12 research articles were found to be eligible for evaluation (longitudinal studies: 1; cross-sectional studies: 11).

Longitudinal studies

Only one prior longitudinal study was identified, reporting that IAF (the parieto-occipital median frequency at 4-13 Hz) exhibited a decrease of 0.55 Hz over a 24-month follow-up period in the dementia due to Alzheimer’s disease (DAD) group [21], a reduction that surpassed the MDC95.

Cross-sectional studies

**Figure 1:**
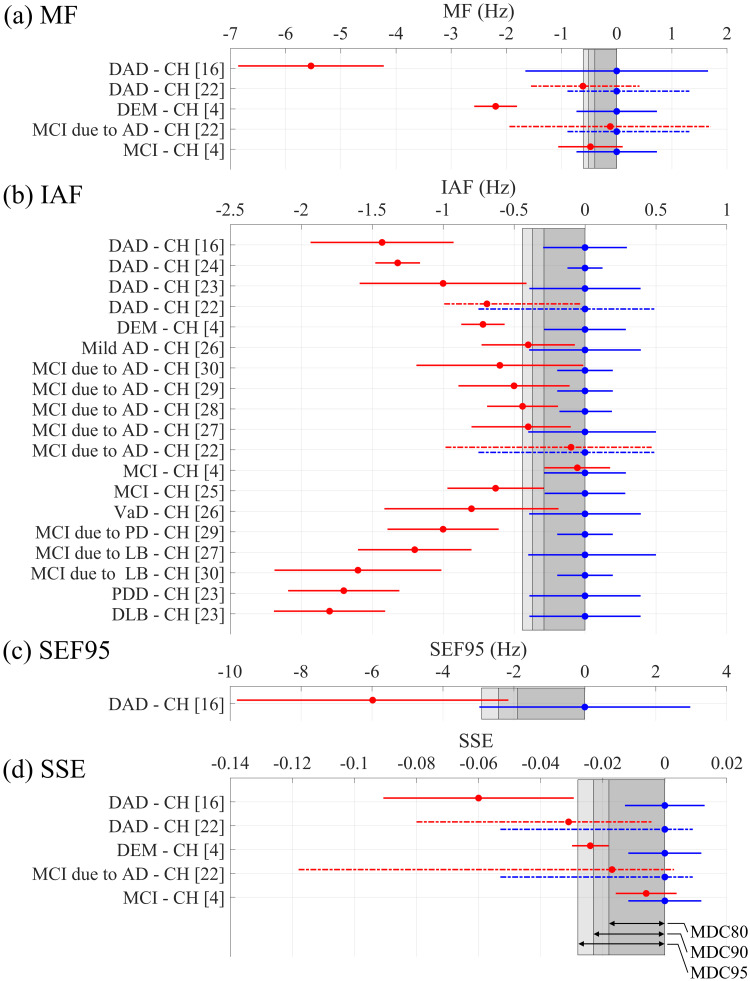
Summary of MDCs and spectral parameters reported in previous cross-sectional studies. The MDCs computed in the present study and group mean/median (corrected for CH group mean/median) with their error range (95% confidence intervals or interquartile range) in the previous studies are summarised for (a) MF, (b) IAF, (c) SEF95, and (d) SSE. A dot indicates either mean (when accompanied by a horizontal solid line indicating 95% confidence intervals) or median (when accompanied by a horizontal dashed line with interquartile range); blue denotes CH and red represents patient groups. The shaded gray areas indicate the ranges of MDC80, MDC90, and MDC95. [4] Hoshi et al., 2022; [16] Poza et al., 2007; [22] Ruiz-Gómez et al., 2018; [23] Babiloni et al., 2017a; [24] de Waal et al., 2013; [25] Garcés et al., 2013; [26] Moretti et al., 2004; [27] Schumacher et al., 2020; [28] Choi et al., 2023; [29] Babiloni et al., 2017b; [30] Babiloni et al., 2018. CH = cognitively healthy; AD = Alzheimer’s disease; PD = Parkinson’s disease; LB = Lewy bodies; DAD = dementia due to Alzheimer’s disease; DEM = dementia; PDD = Parkinson’s disease dementia; DLB = dementia with Lewy bodies; VaD = vascular dementia; MCI = mild cognitive impairment; MF = median frequency; IAF = individual alpha frequency; SEF95 = spectral edge frequency 95; SSE = Shannon’s spectral entropy; MDC = minimal detectable change; MDC95 = MDC at 95% confidence interval; MDC90 = MDC at 90% confidence interval; MDC80 = MDC at 80% confidence interval

Among the 11 previous cross-sectional studies, three studied MF and SSE, 11 studied IAF, and and studied SEF95. In the MF studies, patients with dementia demonstrated values that were smaller than those for CH individuals at 2.19 Hz [4] (Table 2, Figure 1a), surpassing MDC95. In contrast, DAD exhibited values smaller than CH at 0.61 [22] and 5.5 Hz [16], exceeding MDC95. Mild cognitive impairment (MCI) showed smaller values than CH at 0.48 Hz [4] (exceeding MDC80), while MCI due to Alzheimer’s disease (AD) revealed smaller values than CH at 0.12 Hz [22] (below MDCs) [4]. Regarding IAF, dementia exhibited smaller values than CH at 0.72 Hz [4] (Table 2, Figure 1b), which were above MDC95. DAD showed smaller values than CH at frequencies of 0.69 [22], 1.00 [23], 1.32 [24], and 1.43 Hz [16], all surpassing MDC95. Vascular dementia (VaD), Parkinson’s disease dementia (PDD), and dementia with Lewy bodies (DLB) displayed smaller values than CH at 0.80 [21], 1.70 [23], and 1.80 Hz [23], respectively, each exceeding MDC95. MCI showed smaller values than CH at 0.05 [4] (below MDCs) and 0.63 Hz [25] (above MDC95). MCI due to AD and mild AD presented smaller values than CH at 0.10 [22] (below MDCs), 0.40 [26-28] (above MDC90), 0.50 [29], and 0.60 Hz [28] (above MDC95). MCI due to Lewy bodies (LB)/Parkinson’s disease (PD) exhibited smaller values than CH in the range of 1.00-1.60 Hz [27,29,30], exceeding MDC95. For SEF95, DAD showed a smaller value than CH at 5.98 Hz [15] (Table 2, Figure 1c), surpassing MDC95. In the SSE studies, dementia had smaller values than CH at 0.02 [4] (Table 2, Figure 1d), exceeding MDC80, while DAD displayed smaller values than CH at 0.03 [22] and 0.06 [16], each surpassing MDC95. MCI showed a smaller value than CH at 0.01 [4] (below MDCs). MCI due to AD exhibited a smaller value than CH at 0.02 [21], exceeding MDC80.

**Table 2:** Mean/median differences in spectral parameters between groups reported in previous cross-sectional studies. †1 = MEG study; †2 = EEG study. #1 = Sensor space study; #2 = Source space study. *1 = IAF is defined as the frequency which divides PSDn into two equal halves between 4 and 15 Hz. *2 = IAF is defined as the maximum power density peak between 6 and 14 Hz. *3 = IAF represents the frequency with a power peak within the extended alpha range (5–14 Hz). *4 = IAF represents the frequency with a power peak within the extended alpha range (4–13 Hz). *5 = The IAF is defined as follows: (1) all the spectral power values in the 5.5–13 Hz frequency domain were summed and divided by two. (2) The frequency at which the cumulative power in the 5.5–13 Hz band first exceeded the value calculated in step (1) was selected. *6 = IAF is calculated by averaging the peak frequency of a subgroup of occipital channels within the 4–13 Hz frequency range. ‡ = shown as the median difference instead of the mean. CH = cognitively healthy; AD = Alzheimer’s disease; PD = Parkinson’s disease; LB = Lewy bodies; DAD = dementia due to Alzheimer’s disease; DEM = dementia; PDD = Parkinson’s disease dementia; DLB = dementia with Lewy bodies; VaD = vascular dementia; MCI = mild cognitive impairment; MF = median frequency; IAF = individual alpha frequency; SEF95 = spectral edge frequency 95; SSE = Shannon’s spectral entropy; MEG = magnetoencephalography; EEG = electroencephalography

Group comparison	Reference	MF (Hz)	IAF (Hz)	SEF95 (Hz)	SSE	Footnote
DAD – CH	Poza et al., 2007 [16]	-5.54	-1.43	-5.98	-0.06	†1 #1 *1
DAD – CH	Ruiz-Gómez et al., 2018 [22]	-0.61	-0.69		-0.03	†2 #1 *1 ‡
DAD – CH	Babiloni et al., 2017a [23]		-1.00			†2 #1 *2
DAD – CH	de Waal et al., 2013 [24]		-1.32			†2 #1 *6
DEM – CH	Hoshi et al., 2022 [4]	-2.19	-0.72		–0.02	†1 #1 *1
PDD – CH	Babiloni et al., 2017a [23]		-1.70			†2 #1 *2
DLB – CH	Babiloni et al., 2017a [23]		-1.80			†2 #1 *2
VaD – CH	Moretti et al., 2004 [26]		-0.80			†2 #1 *3
Mild AD – CH	Moretti et al., 2004 [26]		-0.40			†2 #1 *3
MCI – CH	Garcés et al., 2013 [25]		-0.63			†2 #2 *4
MCI – CH	Hoshi et al., 2022 [4]	-0.48	-0.05		–0.01	†1 #1 *1
MCI due to AD – CH	Ruiz-Gómez et al., 2018 [22]	-0.12	-0.10		-0.02	†2 #1 *1 ‡
MCI due to AD – CH	Choi et al., 2023 [28]		-0.40			†2 #1 *5
MCI due to AD – CH	Schumacher et al., 2020 [27]		-0.40			†2 #1 *3
MCI due to AD – CH	Babiloni et al., 2017b [29]		-0.50			†2 #1 *2
MCI due to AD – CH	Babiloni et al., 2018 [30]		-0.60			†2 #1 *2
MCI due to PD – CH	Babiloni et al., 2017b [29]		-1.00			†2 #1 *2
MCI due to LB – CH	Schumacher et al., 2020 [27]		-1.20			†2 #1 *3
MCI due to LB – CH	Babiloni et al., 2018 [30]		-1.60			†2 #1 *2

## Discussion

In this study, the within-participant reliability of spectral parameters was estimated in terms of MDC95, MDC90, MDC80, and ICC (Table 1); moreover, it was compared with the observed changes/differences from previous studies (Figure 1, Table 2). Regarding IAF, the MDCs were smaller than both the longitudinal changes and the cross-sectional differences between CH individuals and patients with cognitive impairments from various backgrounds. This indicates that the reported changes/differences are real effects, not attributable to measurement errors. For other spectral parameters (MF, SEF95, and SSE), the MDCs were also smaller than the cross-sectional differences for CH individuals and patients with cognitive impairments, suggesting these differences are meaningful. All spectral parameters showed high ICCs (>0.8), supporting their good within-participant reliabilities. The results are discussed from three perspectives: (1) cognitive and pathological influences on spectral parameters, (2) the impact of measurement errors on these parameters, and (3) the clinical significance of spectral parameters.

Modifying factors of spectral parameters: cognitive impairments and pathologies

The spectral parameters MF, IAF, SEF95, and SSE were shown to be sensitive to the symptoms of cognitive impairments [4]. The parameters were found to be lower in patients with cognitive impairments compared to CH individuals, a phenomenon frequently described as the *slowing* of brain oscillatory activities. This slowing of neuronal oscillatory activities, indicative of the state of neuronal interconnections, likely represents the loss of connections among neuron groups. Although the precise pathological mechanisms underlying this slowing and connection loss remain elusive, several theories have been proposed: first, the connection loss may be attributable to alterations in neurotransmitters, such as a reduction in acetylcholine in patients with DAD [31] and cholinergic dysfunction in patients with DLB/PD [32]; second, a cortico-cortical disconnection might result from the organic loss of pyramidal neurons [33]; and third, the oscillatory slowing could be due to changes in the *generators* of these oscillatory activities [34]. For example, the dominant background rhythm was predominantly within the alpha range in AD, while it tends to be lower (pre-alpha or high theta) in DLB, and the slowing of the dominant EEG rhythm (<8 Hz) assessed visually or through quantitative EEG, which was observed in ~90% of patients with DLB and only ~10% of patients with AD [35]. EEG characteristics of PDD were similar to those of patients with DLB, which supports the hypothesis that PDD and DLB arise from the same spectrum of disease [35]. The mechanism described above suggests that IAF is more likely to be lower in LB/PD than in AD.

Modifying factors of spectral parameters: measurement errors

The changes in the spectral parameters reflect not only cognitive impairments accounted for by pathological changes but also measurement errors, which are usually considered to be biasing factors to be controlled. The nuisance factors that bias the oscillatory signals can be categorized based on their levels: (A) between-participant factors, such as age, gender, personality traits, and other background profiles [17,36,37]; (B) within-participant factors, such as states of vigilance (i.e., drowsiness) [13,14] and mood [38]; and (C) measurement-related factors, including head positions and artifacts [39]. Importantly, these factors demonstrate inclusive relationships; (A) encompasses (B), and (B) encompasses (C). Therefore, the influence of a preceding factor cannot be isolated from the interference of a subsequent one. In this study, we concentrated on the within-participant factors (B), which are influenced by measurement-related factors (C). We quantified the within-participant variabilities using MDCs and ICCs. Concerning (B), the state of vigilance significantly impacts neuronal oscillatory activities. For instance, subjective experiences of sleepiness show a negative correlation with global alpha (8-12 Hz) and a positive correlation with central frontal theta (4-8 Hz) frequencies in resting awake EEG [40]. Participant mood is an additional factor warranting consideration. For example, EEG data indicates an increase in resting-state spectral power density across theta and low-alpha frequency bands, correlating with heightened anxiety and stress levels [38]. Furthermore, the within-participant factors (B) are influenced by measurement-related factors (C). Findings from MEG resting-state oscillatory brain activities suggest that head movement effects are a confounding variable [41].

Our results showed that the MDCs of MF and IAF, which are the gross effects of within-participant (B) and measurement-related factors (C), were <1 Hz, indicating that the effects of within-participant and measurement-related factors can be restricted within 1 Hz when controlled. The repeated measurements were conducted consecutively, with brief intervals between sessions. During these intervals, participants momentarily exited the MEG dewar and remained seated upright in the MEG room for approximately 10 seconds. Given that participants’ moods were unlikely to undergo dynamic changes during these short breaks without specific events (though this was not quantitatively assessed), the primary contributors to the increase in MDCs could be hypothesized as alterations in vigilance state, head position, and the occurrence of transient artifacts. To mitigate potential shifts in vigilance, we instructed participants to exit the dewar and sit upright during breaks, although this technique’s effectiveness in stabilizing vigilance remains unconfirmed. Furthermore, our signal processing pipelines, APTR, aimed at minimizing drowsiness effects, and DSSP and ICA, focused on eliminating transient artifacts, were meticulously designed. Consequently, the MDCs likely represented residual impacts of these nuisance factors, persisting despite quality control measures implemented through standard signal processing methods. Ideally, for enhanced vigilance control, this study should be replicated with measurements spaced out over different times or days rather than consecutively. However, it is important to note that non-consecutive measurements might amplify the influence of mood variations. Interestingly, the ICC was smaller for IAF (0.92) and SSE (0.83) than for MF (0.96) and SEF95 (0.94). MF and SEF95 effectively captured the overall slowing and are thus sensitive to the alterations in the global shape of the PSDn. Conversely, IAF and SSE are sensitive to the local change in PSDn, as the former concentrates on the alpha peak while the latter assesses the irregularity of PSDn (i.e., homogeneity of the spectral distribution). Notably, previous studies have demonstrated that the state of vigilance impacts alpha amplitude [13,14], which explains the lower ICCs in IAF and SSE in detecting local changes in the alpha band of PSDn. This suggests that the state of vigilance may be a significant factor influencing the spectral parameters in repeated measurements.

Clinical significance of spectral parameters

This study computed MDCs for the spectral parameters and evaluated the longitudinal changes and cross-sectional differences reported in the previous studies. Importantly, the MDCs of the spectral parameters were smaller than those observed in the longitudinal/cross-sectional changes/differences detailed in prior studies. This suggests that the impact of pathological factors on the spectral parameters is considerably more pronounced than that of the nuisance factors, bolstering the clinical applicability of these parameters (specifically, MF, IAF, SEF95, and SSE) in monitoring the progression of dementia associated with cognitive impairment over time. Among these four parameters, IAF was the most extensively studied.

The mean difference between patients with MCI due to AD and CH individuals was comparable to MDC95 in IAF, but larger than MDC90 and MDC80. This implies a 5-10% risk (i.e., misattribution of the measurement error) for distinguishing MCI due to AD from CH with IAF in terms of within-subject reliability. In contrast, the inter-rater agreement for physician diagnosis was 70% agreement for no cognitive impairment (i.e., CH individuals), 70% for MCI, and 80% for dementia [42]. Therefore, a reliability of 90-80% is acceptable as a clinical tool. Furthermore, the MDC95 of MMSE is reported to be 5 points [43]. A previous study subjected MMSE to a regression model with each spectral parameter (MF, IAF, and SSE) as a predictor. This analysis revealed that the slopes for MF, IAF, and SSE are 0.298, 0.291, and 0.020, respectively [4]. These slopes estimate the changes in MMSE per unit increase in the spectral parameters. Therefore, a change of 5 points in MMSE would correspond to changes of 1.49 Hz, 1.46 Hz, and 0.10 in MF, IAF, and SSE, respectively. Importantly, this study demonstrated that the MDC95 values for MF, IAF, and SSE are 0.61 Hz, 0.44 Hz, and 0.028, respectively. These values are approximately one-third of the changes corresponding to a 5-point shift in MMSE, suggesting that the spectral parameters have greater reliability than MMSE.

Limitations

This study had two potential limitations. First, this study included comparisons of MDCs with the longitudinal changes in IAF, but not in the other spectral parameters. Preferably, the estimated within-participant variabilities of the spectral parameters (e.g., MDC95) should be evaluated against within-participant changes (i.e., longitudinal changes) in different cognitive statuses (e.g., healthy and with dementia). However, we evaluated most parameters in light of group-level differences reported in previous studies because longitudinal studies were very sparse on this research topic, and to our knowledge, there is only one for IAF [21]. This may have biased our interpretation of the results. Second, participants were limited to younger age groups to avoid hidden disease effects. The variability in the values may differ at different ages. Furthermore, the study involved a limited number of participants (N = 15), with an unbalanced sex distribution (11 females and four males). It is anticipated that future studies will replicate our results with a higher number of participants covering a variety of backgrounds.

## Conclusions

Within-participant variabilities in spectral parameters (i.e., MF, IAF, SEF95, and SSE) were studied in terms of MDC and ICC. These variabilities were smaller than the previously reported differences between healthy individuals and patients with dementia. These results support the clinical use of spectral parameters to examine changes or differences in cognitive status, such as longitudinal monitoring and evaluation of the outcomes of interventions.
